# Integrated deep learning framework for accelerated optical coherence tomography angiography

**DOI:** 10.1038/s41598-022-05281-0

**Published:** 2022-01-25

**Authors:** Gyuwon Kim, Jongbeom Kim, Woo June Choi, Chulhong Kim, Seungchul Lee

**Affiliations:** 1 Department of Mechanical Engineering, Pohang University of Science and Technology (POSTECH), Pohang, 37673 Republic of Korea; 2 Departments of Electrical Engineering and Convergence I.T. Engineering, Medical Device Innovation Center, Pohang University of Science and Technology (POSTECH), Pohang, 37673 Republic of Korea; 3 School of Electrical and Electronics Engineering, College of ICT Engineering, Chung-Ang University, Seoul, 06974 Republic of Korea; 4 Graduate School of Artificial Intelligence, Pohang University of Science and Technology (POSTECH), Pohang, 37673 Republic of Korea

**Keywords:** Imaging and sensing, Biophotonics

## Abstract

Label-free optical coherence tomography angiography (OCTA) has become a premium imaging tool in clinics to obtain structural and functional information of microvasculatures. One primary technical drawback for OCTA, however, is its imaging speed. The current protocols require high sampling density and multiple acquisitions of cross-sectional B-scans to form one image frame, resulting in low acquisition speed. Recently, deep learning (DL)-based methods have gained attention in accelerating the OCTA acquisition process. They achieve faster acquisition using two independent reconstructing approaches: high-quality angiograms from a few repeated B-scans and high-resolution angiograms from undersampled data. While these approaches have shown promising results, they provide limited solutions that only partially account for the OCTA scanning mechanism. Herein, we propose an integrated DL method to simultaneously tackle both factors and further enhance the reconstruction performance in speed and quality. We designed an end-to-end deep neural network (DNN) framework with a two-staged adversarial training scheme to reconstruct fully-sampled, high-quality (8 repeated B-scans) angiograms from their corresponding undersampled, low-quality (2 repeated B-scans) counterparts by successively enhancing the pixel resolution and the image quality. Using an in-vivo mouse brain vasculature dataset, we evaluate our proposed framework through quantitative and qualitative assessments and demonstrate that our method can achieve superior reconstruction performance compared to the conventional means. Our DL-based framework can accelerate the OCTA imaging speed from 16 to 256$\times$ while preserving the image quality, thus enabling a convenient software-only solution to enhance preclinical and clinical studies.

## Introduction

Optical coherence tomography (OCT) is an essential biomedical imaging technique based on low-coherence interferometry to image biological tissues. It enables non-invasive, depth-resolved imaging by demodulating backscattered light interference^1,2^. The ability to resolve axial structure superiorly has led to various clinical applications, such as neurology, ophthalmology, dermatology, gastroenterology, and cardiology^3–5^. Furthermore, OCT has also been extended to functional angiography, namely, optical coherence tomography angiography (OCTA), to study vascular diseases. OCTA extracts the variations in OCT signals caused by the red blood cell (RBC) motion to contrast blood flow within vessels against the static tissue. This functional angiography is accomplished using a sequence of repeated B-scans taken from a single cross-sectional location^6^. The agent-free OCTA modality, owing to its micro-level spatial resolution and non-invasive nature, has enabled anatomical and functional imaging in various clinical applications^7–10^. However, one limitation of OCTA is acquisition speed, bound by the scanning principle employed, i.e., point-by-point raster scanning and the requirement of sequential B-scans. Since high-speed imaging is very desirable for functional imaging, a branch of study has emerged focusing on developing OCT systems with ultrahigh imaging speed, e.g., high-speed optical sources^11–13^ and detection devices^14–16^. However, enahncing imaging speed using hardware adjustments is cost-prohibitive and may pose limitations for practical clinical system implementations.

Recently, several studies have been conducted to enhance the OCTA imaging speed via advances of emerging deep learning (DL)-based methods. Such studies aim to provide software-only solutions that require no modifications to the hardware settings. A study in^17^ adopted convolutional neural network (CNN) architectures to accelerate the B-scan repetition procedure by reconstructing high-quality cross-sectional angiograms (acquired from 48 consecutive B-scans) from a limited number of B-scans (*n* $\le$ 4). For the resulting maximum intensity projection (MIP) *enface* angiograms, the study reported structural similarity index measure (SSIM) and peak signal-to-noise ratio (PSNR) values of 0.63 and 20.82, respectively. Another work in Ref.^18^ conducted pixel-wise super-resolution in the *enface* plane using deep learning to reduce the sampling density while preserving the pixel resolution. Results have outperformed baseline filter-based methods and showed improved signal-to-noise ratio (SNR) and vascular connectivity.

Although DL-based methods have demonstrated the effectiveness in accelerating the OCTA procedure, the following aspects require further investigation, which has motivated the current study. First, all factors related to the imaging speed (i.e., the B-scan repetition number and the sampling density) should be considered simultaneously in a single framework. The previous works^17,18^ addressed only one of the mentioned factors. Secondly, a DL-framework specific to the application should be designed using state-of-the-art DL principles to enhance the desired performance. Previous works, while showed feasibility, mostly utilized already-existing networks designed for other applications (e.g., denoising). Hence, elaborate image-to-image translation techniques (e.g., architecture and loss function design, training strategies) should be considered to establish a framework suited for the current application.

Here we present DL-based principles to accelerate the OCTA acquisition speed while addressing the above limitations. As mentioned before, our work differs from the previous studies in that we aim to tackle all factors related to the OCTA imaging speed (i.e., the B-scan repetition number and the sampling density), and thus enhance the pixel resolution and the image quality in a single integrated framework. By considering different model architectures, loss functions, and training strategies, we establish our networks using a two-staged adversarial training scheme to enhance the pixel resolution and the image quality successively. We evaluate our networks on an in-vivo mouse brain vasculature dataset and conduct a comparative study to select the best-performing model. Finally, we compare quantitative and qualitative results with baseline methods and examine our proposed framework’s reconstruction capability compared to the existing means.

## Materials and methods

In this section, the OCTA dataset acquisition method is briefly described, followed by overall explanations of DL-based methodologies and the proposed approach’s workflow.

**Figure 1:**
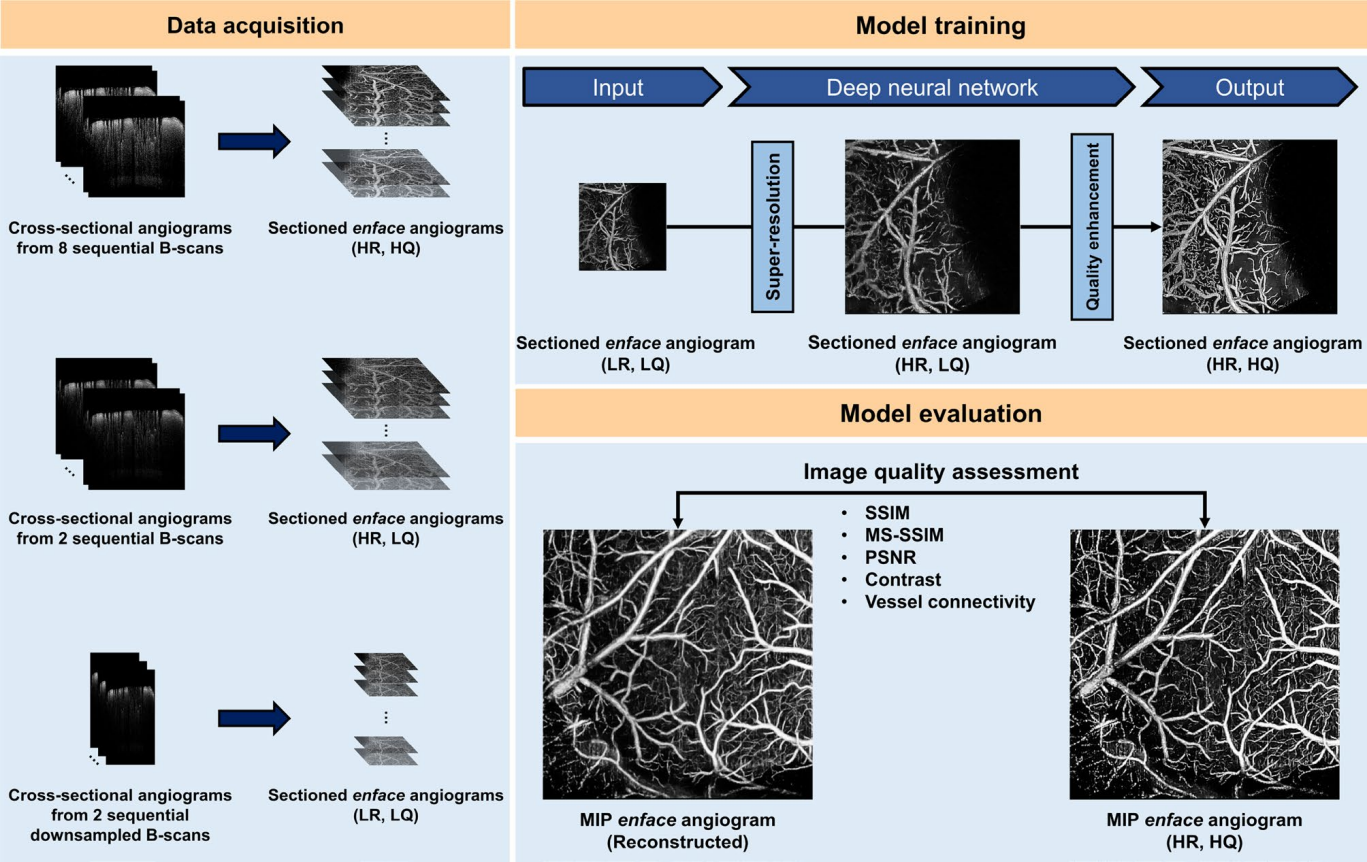
Schematic of our deep learning (DL) framework for accelerated optical coherence tomography angiography (OCTA). *(LR, LQ)* low-resolution and low-quality, *(HR, LQ)* high-resolution and low-quality, *(HR, HQ)* high-resolution and high quality, *SSIM* structural similarity index measure, *MS-SSIM* multiscale structural similarity index measure, *PSNR* peak signal-to-noise ratio.

**Figure 2:**
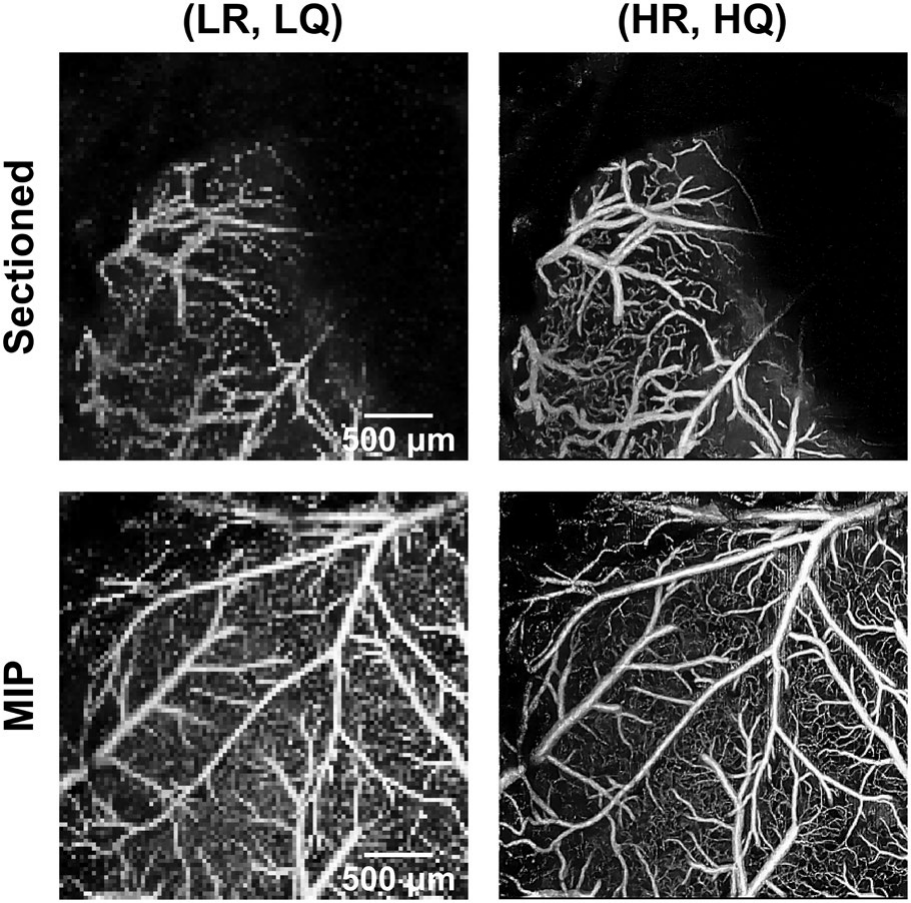
Depiction of how depth-wise sectioned *enface* angiograms differ from maximum intensity projection (MIP) *enface* angiograms. The left column illustrates the low-resolution, low-quality (LR, LQ) angiograms from two repeated downsampled B-scans (downsampling ratio of four). The right column illustrates the corresponding high-resolution, high-quality (HR, HQ) angiograms from eight repeated fully-sampled B-scans.

### Data acquisition

Our dataset comprises in-vivo brain microvasculature images of 8-week-old male mice (C57BL/6J). After removing the scalp and skull (craniotomy details can be found in^7^), the samples were sealed with glass coverslips and imaged using a custom-designed spectral-domain OCT system from the University of Washington Seattle^8^. The system utilizes a broadband superluminescent diode as the light source with a central wavelength of 1340 nm and a 3 dB spectral bandwidth of 110 nm. A biaxial galvanometer is utilized to raster scan the probing light using a scanning step size of 10 $\mu$m, and a fast spectrometer detects the resulting interference signals at an A-line scan rate of 92 kHz. The system’s axial and lateral resolutions are 7 $\upmu$m each, and a 3 mm $\times$ 3 mm field of view (FOV) was utilized. For each volumetric profile, 400 slow-axis locations were sampled at a B-scan rate of 180 Hz, containing cross-sectional structural B-scans with a pixel size of 400 $\times$ 1024 (400 A-lines per scan). As illustrated in Fig. 1, a previously reported OCTA algorithm^7^ was used to register eight sequential B-scans at each slow-axis location to generate a high-quality cross-sectional angiogram. We chose to utilize eight sequential B-scans since it is known that the angiogram quality (e.g., signal-to-noise ratio, vessel connectivity) saturates near eight repeated B-scans^19^. We downsampled the structural volumes in the *enface* plane, and two sequential downsampled B-scans were randomly selected to create a corresponding cross-sectional angiogram with low resolution and quality. The downsampling procedure was conducted to mimic the OCTA systems’ rasterized scanning principle. For example, if the artificial scanning step size is 2$\times$ larger than the fully-sampled step size, we downsample each axis in the *enface* plane by a ratio of two. The fully-sampled angiograms’ *enface* pixel resolution was 400 $\times$ 400, and we investigated downsampling ratios of 2$\times$, 4$\times$, and 8$\times$ in this study. For pre-processing, the pixel values were normalized in the range of [0, 1] to accelerate the deep neural network (DNN) training. In total, 29 pairs of volumetric angiograms were acquired from 11 different biosamples. The acquired volumetric angiograms were sectioned in 2D slices along the depth direction as *enface* representations. The *enface* angiograms from two sequential downsampled B-scans are low in resolution and quality. They are denoted as (LR, LQ) for the remainder of the paper and used as the input data to our DNN framework. Similarly, the high-resolution, high-quality *enface* angiograms from eight sequential fully-sampled B-scans are denoted (HR, HQ) and used as the target for training and ground truth for evaluation. (LR, LQ) and (HR, HQ) angiograms were paired according to the downsampling ratio.

The MIP *enface* angiograms are conventionally utilized to visualize the vascular profile in angiography applications. However, the number of volumetric angiograms available in this study is limited, leading to an insufficient amount of MIP data (29 total) to establish our DNNs. To address the limited data issue, we used 2000 depth-wise sectioned *enface* angiograms from seven volumetric pairs as our training and validation data which were randomly split at a ratio of 9:1. As illustrated in Fig. 2, sectioned *enface* angiograms differ from MIP *enface* angiograms in that void spaces are common in the sectioned *enface* angiograms. Thus, we manually selected images with sufficient vessel profiles to use as our training and validation data. The training dataset was used for optimizing the stochastic gradient descent algorithm, and the validation dataset was used during training to monitor the loss value and finetune the hyper-parameter settings. Random rotation and flipping in the *x* and *y* axes were applied to augment the training dataset. As for the test data, 200 sectioned *enface* angiograms were manually selected from an independent volumetric pair to conduct a comparative study and select the best-performing method. Furthermore, 21 MIP *enface* angiograms from the remaining volumetric pairs were utilized to evaluate the selected methods. A summary description of the dataset is provided in Table 1.

**Table 1:** The in-vivo mouse brain vasculature dataset used in this study. MIP, maximum intensity projection.

	Training/Validation	Test	
Number of volumetric pairs	7	1	21
Image type	Sectioned *enface*	Sectioned *enface*	MIP *enface*
Image quantity	1800/200	200	21

### Deep learning architectures

**Figure 3:**
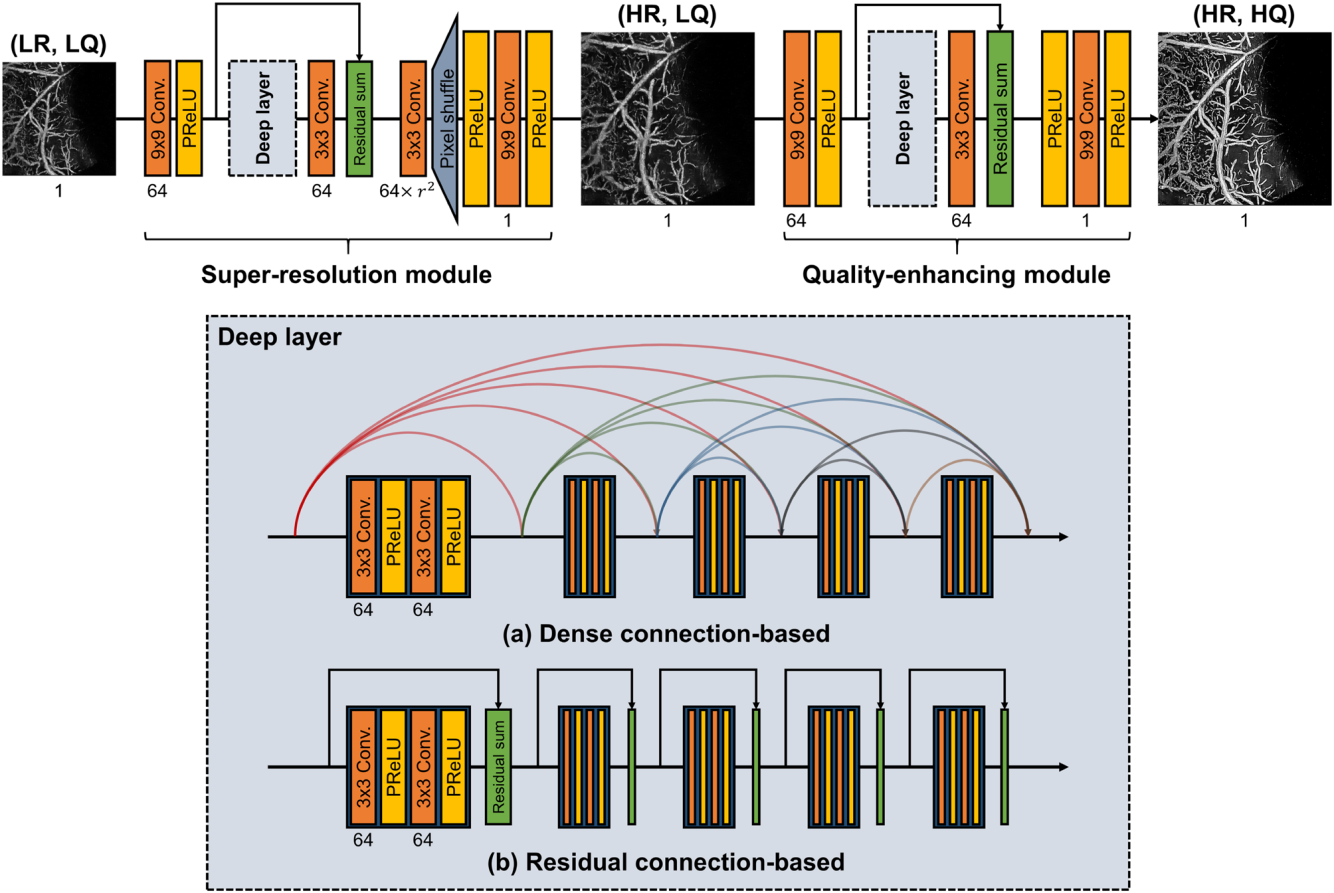
Architectural description of the deep learning (DL) models used in this study: a dense connection-based model (**a**) and a residual connection-based model (**b**). *(LR, LQ)* low-resolution and low-quality, *(HR, LQ)* high-resolution and low-quality, *(HR, HQ)* high-resolution and high quality, *PReLU* parametric rectified linear unit.

We have developed an end-to-end CNN framework to reconstruct (HR, HQ) *enface* angiograms from their (LR, LQ) counterparts. As shown in Fig. 3, our reconstruction model is structured in two-stages and contains two constituent modules that operate in series, i.e., the super-resolution module followed by the quality-enhancing module. The super-resolution module compensates the pixel undersampling, and the quality-enhancing module compensates the B-scan repetition number. Our goal is to establish an end-to-end framework consisting of two sub-modules performing each task successively. The two-stage design should and has in our experiments been found to demonstrate better reconstruction performance than directly mapping (LR, LQ) angiograms to their (HR, HQ) counterparts. Further details on the proposed structure of the networks are summarized in Supplementary Table 1.

The super-resolution module first upsamples the pixel resolution of the undersampled, low quality angiograms (LR, LQ) to their fully-sampled, low-quality counterparts (HR, LQ). Note that (LR, LQ) and (HR, LQ) angiograms are constructed from two repeated B-scans and, therefore, of poor quality. Thus, the task of mapping (LR, LQ) to (HR, LQ) angiograms is conducting pixel-wise super-resolution to fill in the missing pixels from undersampling. The module first extracts low-level features from the input angiograms using (9 $\times$ 9) convolution filters and then extracts high-level residuals using a series of repeated operations within the *Deep layer*. The *Deep layer* consists of five convolution blocks with (3 $\times$ 3) convolution filters and the parametric rectified linear unit (PReLU) activation function^20^. Convolution operations with a smaller receptive field are utilized to capture finer details of the input feature maps, and the PReLU function is employed as our primary activation function since it is known to accelerate deeper networks’ training by incorporating a trainable leakage coefficient^20^.

Two types of inter-block connections within the *Deep layer* are adopted: the dense connection^21^ (Fig. 3a) and the residual connection^22^ (Fig. 3b). We have adopted these inter-connection strategies as they have presented promising results for various previously reported bio-imaging applications^17,18,23^. The dense connection concatenates all subsequent convolution blocks’ output with the original input and utilizes the result as the input to the next convolution block. This configuration strengthens feature propagation since the convolution blocks only need to learn refinements that augment the previous outputs. The residual connection sums the input with the convolution block’s output, which is advantageous as the learning is alleviated to focus on the residuals. Also, these inter-block connections prevent the vanishing gradient problem and reduce the number of parameters for deeper networks. The extracted low-level and high-level features are merged using an element-wise summation operation. For the upsampling layer, we utilize the pixel shuffle operation^24^ to avoid the checkerboard artifacts that are commonly observed when using transposed convolutions. Unlike the previous approach, where the input image is pre-upsampled using filter-based methods^18,23^, we let the network learn the upsampling filters directly to increase performance^25^.

The quality enhancing module enhances the fully-sampled, low-quality output from the previous module and reconstructs the fully-sampled, high-quality angiograms (HR, HQ). The module specifically compensates for the B-scan repetition number (from two repeated B-scans to eight) and thus enhances the angiograms quality. It is worth mentioning that the feature map size is preserved throughout the module as the input and the ground truth (HR, HQ) angiograms are spatially aligned. For the most part, the input and the ground truth angiograms share similar low-frequency information but differ in the high-frequency details. Thus, we designed our module with (9 $\times$ 9) convolution operation to extract the low-frequency information. The operations within the *Deep layer* focus on reconstructing the high-frequency residuals. The two components are merged using the element-wise summation operation. Finally, a 400 $\times$ 400 reconstructed angiogram is obtained through a (9 $\times$ 9) convolution at the end of the module.

As mentioned before, the modules are concatenated in series to form a single reconstruction network. We establish two comparative network models based on the inter-block connection type adopted in the *Deep layer*: the DenseReconstNet (dense connection) and the ResReconstNet (residual connection).

### Model training and evaluation

Several training strategies were employed to train our networks and optimize their trainable parameters. For the loss function *L*, we diverge from the de facto mean squared error (MSE) loss used in the previous studies^17,18^ and instead propose a perceptually improved loss function by combining different loss terms. While the MSE loss achieves high PSNR, minimizing the MSE loss encourages reconstructing pixel-wise averages of plausible outcomes, typically over-smooth and thus failing to capture the high-frequency details of small vessel profiles^25^. We utilize the mean absolute error (MAE) loss as the primary loss function since it is known to have better local convexity^26^:

1 $$\begin{aligned} L^{MAE} = \frac{1}{N} \sum _{i=1}^{N} \left| Y-{\hat{Y}}\right| . \end{aligned}$$

*N* denotes the number of pixels in each angiogram, *Y* and ${\hat{Y}}$ denotes the ground truth and the reconstructed angiogram, respectively. In addition to the primary loss function, we incorporate two other loss terms to aid in reconstructing the high-frequency details:

2 $$\begin{aligned} L^{MS-SSIM} = 1-MSSSIM(Y, {\hat{Y}}), \end{aligned}$$

3 $$\begin{aligned} L^{FMAE} = \frac{1}{N} \sum _{i=1}^{N} \left| \left| F(Y)\right| -\left| F({\hat{Y}})\right| \right|. \end{aligned}$$

The first term is the multiscale structural similarity (MS-SSIM^27^) loss, with the $MSSSIM(\cdot )$ function calculating the corresponding metric. The MS-SSIM loss is advantageous in that it adeptly preserves the contrast in high-frequency regions when utilized along with the MAE loss^26^. The second term is the Fourier MAE loss (FMAE), which calculates the MAE from the magnitude of the angiograms’ 2D Fourier transforms. We choose FMAE to provide information on the vessel orientations and perceive the downsampling remnants as frequency corruptions^23^. The general strategy in incorporating different loss terms is that the primary loss function reconstructs the overall structure and the angiograms’ low-frequency components. In contrast, the two sub-loss terms reconstruct the high-frequency details and remove the angiograms’ frequency-space artifacts. In total, our loss function *L* is designed by linearly combining counterparts and is defined as follows:

4 $$\begin{aligned} L = L^{MAE} + 0.8 \times L^{MS-SSIM} + 7 \times 10^{-5} \times L^{FMAE}. \end{aligned}$$

All the terms included in our loss function are fully differentiable, which is necessary for the neural network’s backpropagation algorithm. The weighting values for the different loss terms were referenced from previous literature^23,26^ and finetuned by heuristic searching and were found to be sufficient for all of the tested models. We mention that our proposed loss function should and has in our experiments been found to provide improved reconstruction performance and training stability than solely using the MAE loss. Each module is sequentially trained during each training iteration. The super-resolution module is trained first, followed by the quality enhancing module using the previous module’s output. At the end of each iteration, the two modules are concatenated as an end-to-end network and finetuned using a lower learning rate. Trainable parameters were initialized using the He normal initialization method^20^ and optimized using the Adam optimizer^28^. Early stopping and L2 regularization^29^ was also employed to avoid over-fitting the network parameters.

**Figure 4:**
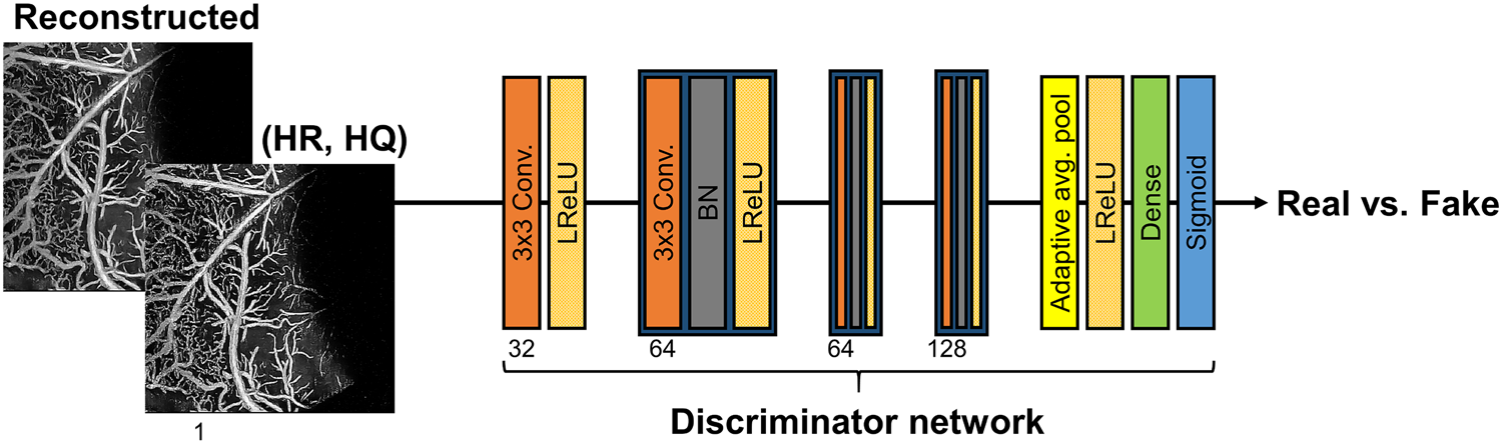
Architectural description of the discriminator network used for the adversarial training. (HR, HQ), high-resolution and high-quality; LReLU, leaky rectified linear unit; and BN, batch normalization.

After successfully establishing the DenseReconstNet and the ResReconstNet, we further trained each network using an adversarial training scheme to boost the reconstruction performance. As illustrated in Fig. 4, we define a discriminator network *D*, which we optimized in an alternating manner with our pre-trained reconstruction model *G* to solve the adversarial min-max problem^30^:

5 $$\begin{aligned} \min \limits _{G} \max \limits _{D} E_{Y\sim p_{data}(Y)}[logD(Y)] + E_{X\sim p_{data}(X)}[log(1-D(G(X)))]. \end{aligned}$$

*X* denotes the (LR, LQ) angiogram taken as the input to our reconstruction model. The idea is that we train our reconstruction model to fool the discriminator that distinguishes the reconstructed angiograms from their (HR, HQ) counterparts. The proposed training strategy allows our reconstruction model to create perceptually superior solutions residing in the manifold of the (HR, HQ) angiograms. The adversarial loss function for the reconstruction models is defined as follows^30^:

6 $$\begin{aligned} L^{Adv} = L + 5 \times 10^{-3} \times (-logD(G(X))). \end{aligned}$$

The weight value for the adversarial loss term was referenced from previous literature^25^ and finetuned by heuristic searching and was found to be sufficient for all of the tested models. The established generative adversarial networks (GANs) are denoted as DenseReconstGAN and ResRecontGAN. Details on the discriminator network’s structure can be found in Supplementary Table 2. All hyper-parameters were searched for by using the random search strategy^31^ and their details can be found in Supplementary Table 3.

As for the evaluation metric, we consider the perceptual indices of SSIM^32^, MS-SSIM^27^, and the PSNR to assess the reconstruction performance. In addition, we assess the angiograms’ contrast by calculating the root-mean-square (RMS) contrast^33^ and the vessel connectivity by calculating the ratio of connected flow pixels to the total number of skeletonized pixels^18,19^.

## Results and discussion

In this section, we present and discuss the experimental results of our OCTA reconstruction method. We again emphasize that our task differs from image super-resolution in the strict sense because our method compensates for both the pixel undersampling (image resolution) and the B-scan repetition number (image quality). However, no previous work has attempted to address both factors in a single integrated framework. Therefore, we compare our method with existing super-resolution methods, including interpolation-based image upsampling methods (i.e., nearest-neighbor, bicubic, and Lanczos interpolation) and a previously reported DL-based super-resolution network for retinal OCT angiograms called the high-resolution angiogram reconstruction network (HARNet)^18^. Implementation details for HARNet were carefully examined from the previous literature^18^, and its hyper-parameters were optimized using the random search strategy^31^ (Supplementary Table 3). We first quantitatively compare the reconstruction performance of the presented methods using the test dataset consisting of sectioned *enface* angiograms. Once the methods showing the most promising results are identified, we further assess quantitative and qualitative results on the test dataset consisting of MIP *enface* angiograms from various in-vivo samples.

### Quantitative comparison using sectioned *enface* angiograms

To emphasize the advantage of our proposed training strategy (i.e., two-staged training with the perceptual loss function followed by adversarial training), we conduct a full ablation study in addition to the comparative study with the baseline methods, and quantitative results are summarized in Table 2. We mention that although HARNet was originally proposed as a super-resolution framework, it was trained to map (LR, LQ) images to (HR, HQ) images in our experiments and thus learns to compensate for both the pixel undersampling (image resolution) and the B-scan repetition number (image quality). Quality enhancement filters (e.g., median, bilateral, vesselness) were not used for the interpolation-based methods since sectioned *enface* angiograms do not contain full vascular profiles (see Fig. 2), and applying such filters deteriorated the results. For the same reason, angiogram quality measures (i.e., the contrast and vessel connectivity) were not assessed, and only the image reconstruction metrics were considered (i.e., SSIM, MS-SSIM, and PSNR). However, an additional quality enhancement filter was applied for the interpolation-based method when evaluating the MIP *enface* angiograms in the following section, and all metrics were assessed.

**Table 2:** Performance metrics of different methods evaluated on the test dataset composed of sectioned *enface* angiograms.

Ratio	Metric	Baseline				Proposed					
		Interpolation			Deep learning	Single-stage with MAE		Two-stage with MAE		Proposed training strategy	
		Nearest	Bicubic	Lanczos	HARNet^18^	ResNet	DenseNet	ResNet	DenseNet	ResGAN	DenseGAN
*r* = 2	SSIM	0.740 ± 0.031	${0.756 }\pm {0.033 }$	0.751 ± 0.031	${0.794 }\pm {0.023 }$	0.809 ± 0.023	0.824 ± 0.025	0.889 ± 0.031	0.913 ± 0.021	0.910 ± 0.032	${0.921 }\pm {0.021 }$
	MS-SSIM	0.872 ± 0.012	${0.879 }\pm {0.012 }$	0.878 ± 0.010	${0.912 }\pm {0.008 }$	0.923 ± 0.006	0.928 ± 0.009	0.951 ± 0.021	0.972 ± 0.012	0.972 ± 0.011	${0.975 }\pm {0.010 }$
	PSNR [dB]	25.75 ± 3.16	${26.54 }\pm {3.16 }$	26.37 ± 3.14	${27.19 }\pm {2.95 }$	27.35 ± 2.87	27.38 ± 2.83	26.78 ± 3.31	29.07 ± 2.98	28.04 ± 3.20	${29.30 }\pm {2.93 }$
*r* = 4	SSIM	0.706 ± 0.042	${0.720 }\pm {0.040 }$	0.711 ± 0.044	${0.744 }\pm {0.059 }$	0.745 ± 0.060	0.772 ± 0.060	0.780 ± 0.032	0.810 ± 0.041	0.797 ± 0.041	${0.862 }\pm {0.041 }$
	MS-SSIM	0.819 ± 0.021	${0.834 }\pm {0.021 }$	0.831 ± 0.024	${0.852 }\pm {0.040 }$	0.854 ± 0.046	0.863 ± 0.038	0.900 ± 0.014	0.913 ± 0.020	0.910 ± 0.013	${0.937 }\pm {0.021 }$
	PSNR [dB]	23.11 ± 3.46	${23.91 }\pm {3.43 }$	23.76 ± 3.42	${24.05 }\pm {3.33 }$	24.10 ± 3.27	24.27 ± 3.30	25.87 ± 3.36	25.28 ± 3.26	25.85 ± 3.36	${26.00 }\pm {3.24 }$
*r* = 8	SSIM	0.678 ± 0.064	${0.681 }\pm {0.051 }$	0.665 ± 0.053	${0.713 }\pm {0.058 }$	0.725 ± 0.059	0.739 ± 0.062	0.726 ± 0.054	0.726 ± 0.051	0.755 ± 0.062	${0.755 }\pm {0.041 }$
	MS-SSIM	0.743 ± 0.051	${0.762 }\pm {0.052 }$	0.757 ± 0.051	${0.769 }\pm {0.079 }$	0.775 ± 0.080	0.790 ± 0.081	0.833 ± 0.042	0.868 ± 0.031	0.855 ± 0.041	${0.892 }\pm {0.020 }$
	PSNR [dB]	21.12 ± 3.79	${21.80 }\pm {3.73 }$	21.64 ± 3.74	${22.08 }\pm {3.17 }$	22.86 ± 3.14	22.94 ± 3.20	22.88 ± 3.77	24.55 ± 3.17	22.93 ± 3.72	${25.58 }\pm {3.01 }$

Our deep learning models are denoted as ResNet, ResGAN, DenseNet, and DenseGAN in short. *MAE* mean absolute error, *SSIM* structural similarity index measure, *MS-SSIM* multiscale structural similarity index measure, *PSNR* peak signal-to-noise ratio.

The models trained with our proposed training strategy (ResReconstGAN and DenseReconstGAN) outperform all baseline methods, including the interpolation-based methods and the previously reported HARNet. When solely using the MAE loss, the networks trained with the two-staged training scheme show improved performance over those trained in a single-stage manner that directly maps (LR, LQ) angiograms to their (HR, HQ) counterparts, including HARNet. The two-staged training strategy enhances the reconstruction performance since it provides additional intermediate supervision during training to perform super-resolution to (HR, LQ) angiograms. Furthermore, Table 2 shows that training with our proposed perceptual loss function and the adversarial training scheme further boosts the reconstruction performance as the metrics improved over the two-staged networks trained solely using the MAE loss. The results follow the intuition that the sub-loss terms and the adversarial loss push the reconstructed results to the (HR, HQ) angiograms’ manifold. The dense connection-based models (DenseReconstNet and DenseReconstGAN) outperform the residual connection-based models (HARNet, ResReconstNet, and ResReconstGAN), which is consistent with the consensus that deeper networks benefit from their ability to model mappings of higher complexity^34,35^. It is worth mentioning that the previously reported HARNet shows poor reconstruction performance even compared to our single-stage models trained using the MAE loss. One possible reason could be that while it is known that pre-upsampling the input image using filter-based methods deteriorates the results^25^, our networks learn the upsampling filters directly in contrast to HARNet. DenseReconstGAN consistently shows the best performance amongst all comparative methods for each of the downsampling ratios. HARNet shows the best performance amongst the baseline methods, and the bicubic interpolation method outperforms all other interpolation-based methods. Therefore, we focus our analysis on comparing the three methods for assessing the MIP test dataset, and the results are presented in the following section.

### Quantitative and qualitative comparison using MIP *enface* angiograms

**Table 3:** Performance metrics of our DenseReconstGAN, HARNet, and the bicubic interpolation method with bilateral filtering evaluated on the test dataset composed of maximum intensity projection (MIP) *enface* angiograms from various biosamples.

Ratio	Metric	Bicubic + Bilateral	HARNet^18^	DenseReconstGAN
*r* = 2	SSIM	0.518 ± 0.022	0.779 ± 0.057	${0.904 }\pm {0.068 }$
	MS-SSIM	0.862 ± 0.020	0.900 ± 0.044	${0.965 }\pm {0.044 }$
	PSNR [dB]	15.17 ± 0.79	21.23 ± 1.47	${25.40 }\pm {2.31 }$
	Contrast	0.226 ± 0.007	0.266 ± 0.007	${0.270 }\pm {0.007 }$
	Connectivity	0.834 ± 0.014	0.868 ± 0.010	${0.877 }\pm {0.008 }$
	FPS	**93.46**	21.06	21.81
*r* = 4	SSIM	0.281 ± 0.029	0.617 ± 0.061	${0.730 }\pm {0.046 }$
	MS-SSIM	0.618 ± 0.038	0.802 ± 0.058	${0.881 }\pm {0.036 }$
	PSNR [dB]	12.56 ± 0.85	18.34 ± 1.47	${20.21 }\pm {1.44 }$
	Contrast	0.205 ± 0.010	0.254 ± 0.008	${0.264 }\pm {0.009 }$
	Connectivity	0.665 ± 0.040	0.864 ± 0.009	${0.866 }\pm {0.010 }$
	FPS	**96.56**	23.81	23.78
*r* = 8	SSIM	0.188 ± 0.021	0.347 ± 0.065	${0.408 }\pm {0.048 }$
	MS-SSIM	0.356 ± 0.032	0.582 ± 0.068	${0.679 }\pm {0.054 }$
	PSNR [dB]	10.92 ± 0.60	14.89 ± 1.19	${15.86 }\pm {1.15 }$
	Contrast	0.198 ± 0.006	0.216 ± 0.011	${0.245 }\pm {0.009 }$
	Connectivity	0.651 ± 0.029	0.727 ± 0.018	${0.762 }\pm {0.021 }$
	FPS	**99.75**	23.95	21.81

*SSIM* structural similarity index measure, *MS-SSIM* multiscale structural similarity index measure, *PSNR* peak signal-to-noise ratio, *FPS* frames per second.

Significance values are given in bold.

**Figure 5:**
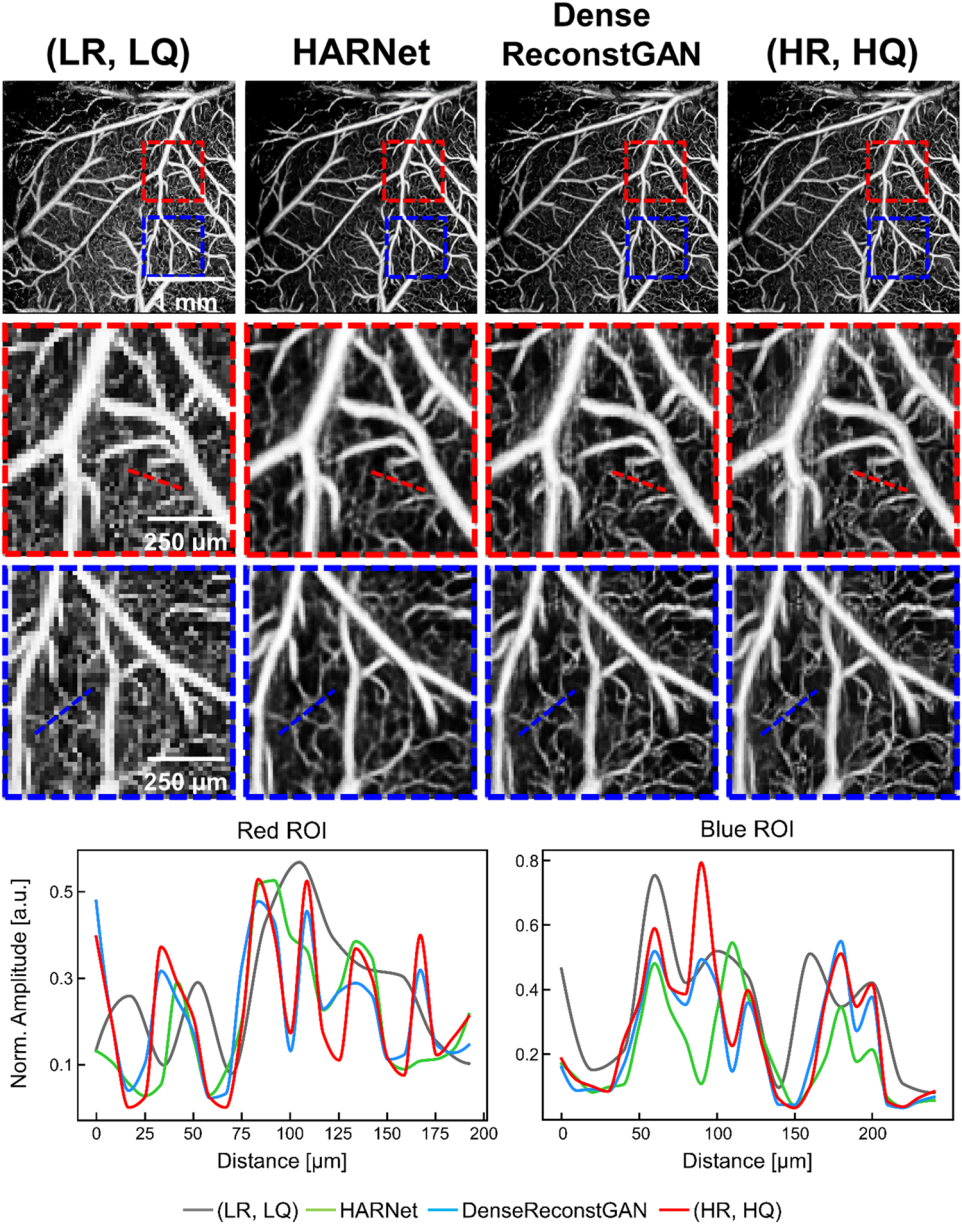
Qualitative performance of our DenseReconstGAN and HARNet for reconstructing at a downsampling ratio of two. Enlarged profiles of the boxed regions of interest (ROI) are illustrated. Vessel profiles along the dashed lines within each ROI are also illustrated. *(LR, LQ)* low-resolution and low-quality, *(HR, HQ)* high-resolution and high quality.

**Figure 6:**
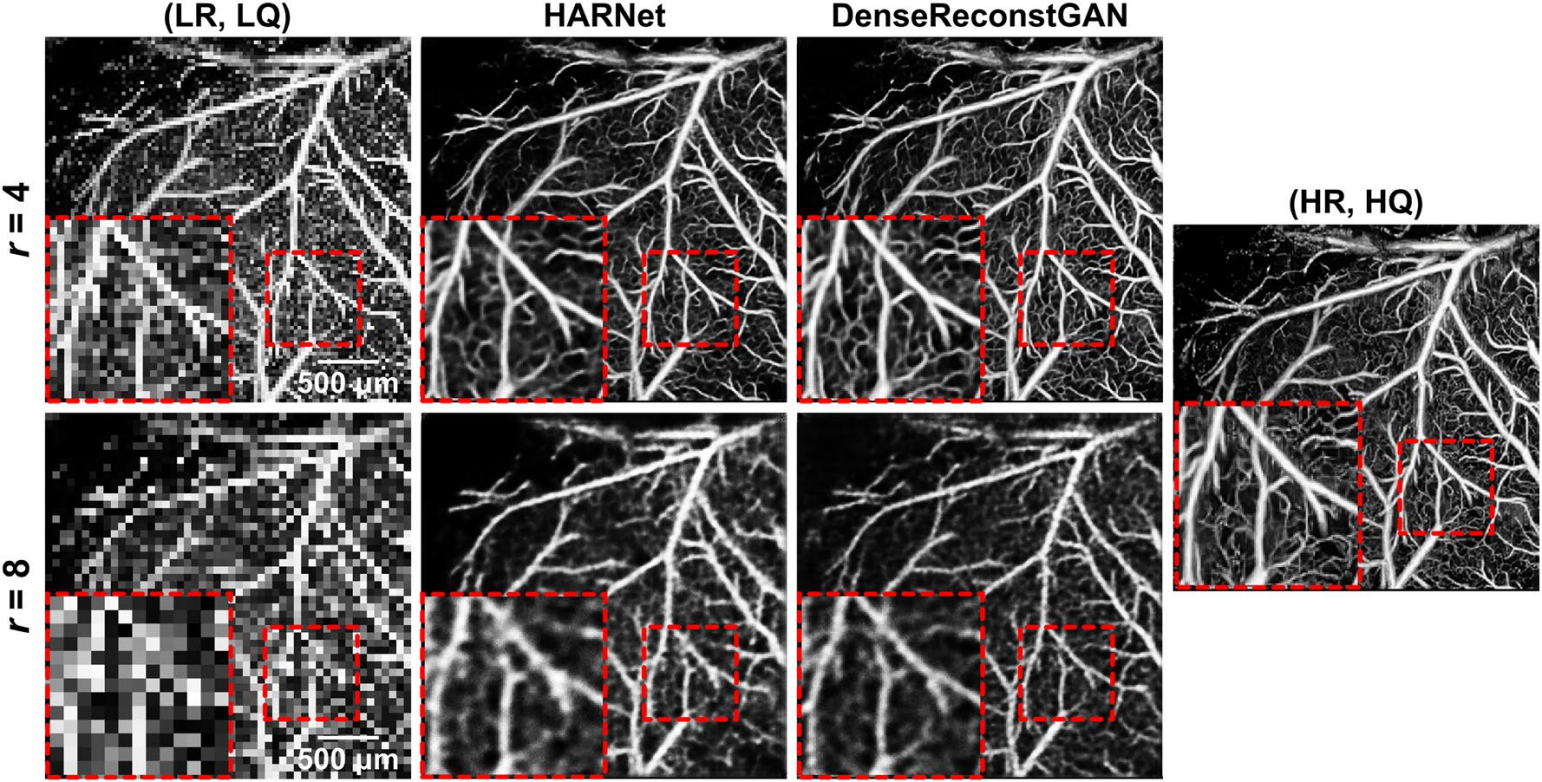
Qualitative performance of our DenseReconstGAN and HARNet for reconstructing at downsampling ratios (denoted as *r*) of four and eight. *(LR, LQ)* low-resolution and low-quality, *(HR, HQ)* high-resolution and high quality.

We compare the performance of the DenseReconstGAN, HARNet, and the bicubic interpolation method for the test dataset consisting of MIP *enface* images taken from various biosamples. The quantitative results of the three comparison methods are presented in Table 3. A bilateral filter was applied to the bicubic interpolation results to enhance the angiogram quality for fair comparisons with the DL-based methods. The results indicate that our DenseReconstGAN is superior to the baseline methods in terms of image reconstruction measures (i.e., SSIM, MS-SSIM, and PSNR) and angiogram quality measures (i.e., the contrast and vessel connectivity) for all downsampling ratios. Also, the results show that our DNN, trained with sectioned *enface* images, can generalize to full vascular profiles in MIP *enface* images. However, we notice that all evaluation metrics using the MIP *enface* dataset are lower than the sectioned *enface* dataset (see Table 2), especially for higher downsampling ratios of four and eight. The main reason is that the void regions in the sectioned *enface* angiograms yield high evaluation metrics, for they do not include contents for reconstruction (see Fig. 2). In addition, assessing the MIP *enface* angiograms require higher generalizability, as they are taken from various in-vivo samples. Nevertheless, our DenseReconstGAN still exhibits notable metrics at downsampling ratios of two and four and significantly outperforms the baseline methods, including the previously reported HARNet, which confirms the superiority of our proposed DL-based framework.

Figure 5 illustrates the qualitative performance of our DenseReconstGAN and HARNet on an (LR, LQ) MIP *enface* angiogram with a downsampling ratio of two. In the enlarged regions of interest (ROI), it can be seen that HARNet suffers from contrast loss and oversmooths the vascular details, resulting in dilated vessel structures. In contrast, our DenseReconstGAN demonstrates improved reconstruction precision with obvious advantages in recovering the high-frequency microvascular details owing to our improved training strategy. Since capillary morphology provides critical information about vascular pathology (e.g., capillary telangiectasias), we specifically compare the reconstruction performance of very small vessels ($\sim$ 50 $\upmu$m in diameter) along the dashed lines within each ROI. It can be observed that the input (LR, LQ) angiogram exhibits distorted and out-of-phase vessel profiles due to spatial aliasing. HARNet fails to distinguish vessels of smaller diameters (vessel profiles in the distance range of 100–125 $\upmu$m, 150–175 $\upmu$m for the red ROI, and 75–100 $\upmu$m blue ROI) and severely loses contrast (0–25 $\upmu$m red ROI and 150–200 $\upmu$m blue ROI). Conversely, DenseReconstGAN reconstructs in-phase microvascular profiles with relatively small contrast loss. In addition, the vessel profiles indicate that our DenseReconstGAN produces no false blood flow signals. These results are better reflected in the angiograms’ contrast and vessel connectivity values in Table 3, as DenseReconstGAN shows superior values for all downsampling ratios. Some minor limitations can be observed using our method in certain poorly sampled regions. For example, local smoothing (115–150 $\upmu$m red ROI) is observed in regions where spatial aliasing is most severe in the input (LR, LQ) angiogram. In addition, DenseReconstGAN also suffers from wavy motion artifacts, as can be observed in the red ROI. Our DL model is more prone to these artifacts since high expression capability, unfortunately, enhances noise. Our framework does not make efforts to correct these artifacts since commercial systems can easily address these artifacts by tracking the scan acquisition level and utilizing related software as described in Refs.^36,37^. Despite the above limitations, the qualitative results indicate that our proposed DL framework exhibits significantly superior performance than the bicubic interpolation method with bilateral filtering and the previously reported HARNet in reconstructing the microvascular profiles.

We present a qualitative comparison of the two methods at higher downsampling ratios of four and eight (Fig. 6). A clear distinction can be made regarding the quality of *vesselness*, as the difference between the two methods becomes more significant with sparse data. At a downsampling ratio of four, oversmoothing and contrast loss worsens for HARNet, whereas microvascular structure and contrast are reasonably preserved for our DenseReconstGAN. Similarly, at a downsampling ratio of eight, HARNet exhibits biologically dubious features (jagged and disjointed vessels), while our DenseReconstGAN reconstructs smoother and rounder vessel profiles. However, at such a high downsampling ratio, the performance of our DL model deteriorates severely. Since the downsampling ratio of eight is equivalent to a scanning step size of 80 $\upmu$m, and the scanning step size far exceeds the maximum Nyquist limit required to capture the capillary resolution ($\sim$ 10 $\upmu$m^38^), the degraded results are expected. Thus, the desired application context should always be considered when selecting the appropriate scanning step size to meet the desired performance. For example, a large scanning step size (e.g., 40 $\upmu$m) is more appropriate for clinical applications requiring fast imaging speed but tolerant to the low image quality. Nevertheless, our DL framework still outperforms the previously reported super-resolution network HARNet in reconstructing the vasculature’s bulk physiology when using larger scanning step sizes of 40 and 80 $\upmu$m. Thus, our method expands the range of usable scanning step sizes in OCTA systems, which were considered unavailable previously.

We also compare the computation time and the mean frames per second (FPS) rate is provided in Table 3 (CPU and GPU details are listed in Supplementary Table 3). Even though our DL model is very deep with a high parameter dimension, it achieves a reasonably fast computation time during inference ($\sim$ 23.78 FPS) because the forward path of a trained DNN model comprises only basic operations and nonlinear activation functions. HARNet exhibits a similar inference speed ($\sim$ 23.95 FPS) since it is also a very deep model with a similar parameter dimension. Although the bicubic interpolation method with bilateral filtering offers the fastest computation time ($\sim$ 99.75 FPS), it shows the worst reconstruction performance. Our DenseReconstGAN shows the best reconstruction results while offering computation speed sufficient for real-time embedded software usage. The computation time does not increase with the downsampling ratio, indicating that its consideration can be ignored when choosing the scanning step size. While (HR, HQ) OCTAs’ mean acquisition time was 17.8 s, the acquisition time reduced significantly for the (LR, LQ) OCTA case. For example, doubling the scanning step size (*r* = 2) and using two sequential B-scans would result in a 16$\times$ faster acquisition time, i.e., approximately 1.1 s. Similarly, ratios of four and eight would result in 64$\times$ and 256$\times$ faster acquisition time, i.e., 0.28 and 0.07 seconds, respectively. Thus, we believe our DL-based reconstruction framework is superior to the previously reported methods as it significantly reduces the (HR, HQ) OCTA’s acquisition time ($\le$ 1.2 s) while presenting enhanced reconstruction performance.

## Conclusions

This study proposed a deep learning-based software-only solution to accelerate the OCTA systems’ imaging speed while preserving the image quality. The main contributions of the work are summarized as follows. First, we tackled all existing inherent factors related to the slow OCTA imaging speed, including the B-scan repetition number and the sampling density. While previous works only partially addressed the limiting factors, our work considered all aspects in a single, end-to-end framework. As a result, our approach achieved an acceleration of OCTA imaging by factors of up to 16–256$\times$. Our method is advantageous since it can be directly applied to clinically available systems without increasing the system complexity. In addition, larger FOVs can be captured to improve clinical observations without sacrificing spatial resolution. Second, we established a novel DNN framework by leveraging state-of-the-art deep learning principles (i.e., network architectures, loss functions, and training strategies). In particular, we showed that the DenseReconstGAN exhibited the best performance and significantly outperformed the baseline methods, including the previously reported HARNet, in both quantitative and qualitative aspects for all of the experimented cases (Tables 2, 3, Figs. 5, 6). The results indicate that our proposed DNN framework is more suitable for the current application of compensating both the B-scan repetition number and the sampling density.

In the future, we aim to continually refine our DL framework’s generalizability by training with more MIP *enface* images from various in-vivo samples. We also aim to extend our framework to other pathological applications (e.g., retinal angiography). By combining our established framework with transfer learning techniques^39^, we can avoid acquiring a large amount of data that is required for re-training. We hope to utilize our integrated framework to broaden OCTA systems’ clinical applicability to rapidly and accurately delineate pathological biomarkers and achieve potential near real-time functional imaging capability.

## Supplementary Information

Supplementary Tables.
