# Supplementary material for: Integrated deep learning framework for accelerated optical coherence tomography angiography
Source: Sci Rep. 2022 Jan 25;12:1289. doi: 10.1038/s41598-022-05281-0 (PMC8789830; doi:10.1038/s41598-022-05281-0)
Supplement: Supplementary file 1 — Supplementary Tables. [file 41598_2022_5281_MOESM1_ESM.docx]

Supplementary Materials for:

Integrated Deep Learning Framework for Accelerated Optical Coherence Tomography Angiography

Gyuwon Kim^a,1^, Jongbeom Kim^a,b,1^, Woo June Choi^c,*^, Chulhong Kim^a,b,d*^, and Seungchul Lee^a,d,*^

^a.^ *Department of Mechanical Engineering, Pohang University of Science and Technology (POSTECH), Pohang 37673, Republic of Korea*

^b.^ *Departments of Electrical Engineering and Convergence IT Engineering, Medical Device Innovation Center, Pohang University of Science and Technology (POSTECH), Pohang 37673, Republic of Korea*

^c^**^.^** *School of Electrical and Electronics Engineering, College of ICT Engineering, Chung-Ang University, Seoul 06974, Republic of Korea*

^d^**^.^** *Graduate School of Artificial Intelligence, Pohang University of Science and Technology (POSTECH), Pohang 37673, Republic of Korea*

^1^Gyuwon Kim and Jongbeom Kim contributed equally to this work.

*Corresponding author: Seungchul Lee

E-mail: [seunglee@postech.ac.kr](mailto:seunglee@postech.ac.kr)

*Corresponding author: Chulhong Kim

E-mail: chulhong@postech.edu

*Corresponding author: Woo June Choi

E-mail: cecc78@cau.ac.kr

Supplementary Table 1. Structural information of the deep learning (DL) models used in this study. The number of output channels is preserved after the parametric rectified linear unit (PReLU) operations. *r* denotes the downsampling ratio.

| **Super-resolution module** | | | | | | **Quality-enhancing module** | | | | | |
| --- | --- | --- | --- | --- | --- | --- | --- | --- | --- | --- | --- |
| Layer | Operation | Channels | Filter size | Stride | Padding | Layer | Operation | Channels | Filter size | Stride | Padding |
| Conv1 | Convolution | 64 | 9$\times$9 | 1 | 4 | Conv1 | Convolution | 64 | 9$\times$9 | 1 | 4 |
| Deep layer* | Convolution | 64 | 3$\times$3 | 1 | 1 | Deep layer* | Convolution | 64 | 3$\times$3 | 1 | 1 |
|  | Convolution | 64 | 3$\times$3 | 1 | 1 |  | Convolution | 64 | 3$\times$3 | 1 | 1 |
|  | Concat./  Residual sum. | 64$\times$*n/*  64 | - | - | - |  | Concat./  Residual sum. | 64$\times$*n/*  64 | - | - | - |
| Conv2 | Convolution | 64 | 3$\times$3 | 1 | 1 | Conv2 | Convolution | 64 | 3$\times$3 | 1 | 1 |
| Residual sum. | Residual sum. | 64 | - | - | - | Residual sum. | Residual sum. | 64 | - | - | - |
| Conv3 | Convolution | 64$\times r^{2}$ | 3$\times$3 | 1 | 1 | Conv3 | Convolution | 1 | 9$\times$9 | 1 | 4 |
| Upsample | Pixel shuffle | 64 | - | - | - |  | | | | | |
| Conv4 | Convolution | 1 | 9$\times$9 | 1 | 4 |  |  |  |  |  |  |

* Operations included in the *Deep layer* are repeated five times, as illustrated in Fig. 3. The concatenation operation is conducted for the dense connection-based models with 1$\leq n\leq$5, while the residual summation operation is conducted for the residual connection-based models.

Supplementary Table 2. Structural information of the discriminator network used for the adversarial training. The number of output channels is preserved after the leaky rectified linear unit (LReLU) and batch normalization (BN) operations. The leakage coefficient of the LReLU activation function is set to 0.2.

| **Discriminator network** | | | | | | |
| --- | --- | --- | --- | --- | --- | --- |
| Layer | Operation | Channels | Filter size | Stride | Padding | Neurons |
| Conv1 | Convolution | 32 | 3$\times$3 | 1 | 1 | - |
| Conv2 | Convolution | 64 | 3$\times$3 | 2 | 1 | - |
| Conv3 | Convolution | 64 | 3$\times$3 | 2 | 1 | - |
| Conv4 | Convolution | 128 | 3$\times$3 | 2 | 1 | - |
| Pool1 | Adaptive avg. pooling | - | - | - | - | 128 |
| Dense1 | Dense | - | - | - | - | 1 |

Supplementary Table 3. Training settings of the deep learning (DL) models used in this study. Network models are implemented using Python 3.6.8 with a PyTorch backend. Model training and evaluation are conducted using an NVIDIA TITAN Xp GPU and an Intel®CoreTM i5-8400 x64 16.0GB RAM CPU.

| **Training settings** | | | | |
| --- | --- | --- | --- | --- |
| Network | Module | Learning rate | Batch size | Weight decay |
| ReconstNet | Super-resolution module | ${10}^{-4}$ | 12 | $2\times{10}^{-5}$ |
|  | Quality-enhancing module | ${10}^{-4}$ | 12 | $2\times{10}^{-5}$ |
|  | Reconstruction network | ${10}^{-5}$ | 12 | $2\times{10}^{-5}$ |
| ReconstGAN | Reconstruction network | $5\times{10}^{-6}$ | 12 | $2\times{10}^{-5}$ |
|  | Discriminator | ${10}^{-3}$ | 12 | $2\times{10}^{-5}$ |
| HARNet^1^ | Reconstruction network | ${10}^{-4}$ | 12 | $2\times{10}^{-5}$ |

REFERENCES

1. Gao, M. *et al*. Reconstruction of high-resolution 6x6-mm OCT angiograms using deep learning. *Biomed. Opt. Express* **11**, 3585–3600 (2020).
